# Measurement of Hair Cortisol Throughout Gestation

**DOI:** 10.3390/jcm15083052

**Published:** 2026-04-16

**Authors:** Jusselit Estrada, María Angélica Miglino, Nikol Ponce-Rojas, Mariano del Sol

**Affiliations:** 1 Escuela de Obstetricia, Facultad de Ciencias para el Cuidado de la Salud, Universidad San Sebastián, Concepción 4081339, Chile; 2 Faculty of Medicine, Doctoral Program in Morphological Sciences, University of La Frontera, Temuco 4780000, Chile; nikol.ponce@ufrontera.cl; 3 Veterinary Medicine, Universidade de Marília (Unimar), Marília 17525-902, Brazil; miglino@unimar.br; 4 Center of Excellence in Surgical and Morphological Studies (CEMyQ), Universidad de La Frontera, Temuco 4811230, Chile

**Keywords:** cortisol, methods, hair, pregnancy, chemiluminescence

## Abstract

**Background/Objectives**: Cortisol has become established as a relevant biomarker due to its association with various pathologies, including its potential utility in mental health research. However, regarding the techniques employed for its analysis, the available literature shows a certain degree of heterogeneity both in the methods used to obtain cortisol and in the analytical techniques employed for its measurement. This makes it difficult to compare results across specific populations, particularly in pregnant women, who experience metabolic and physiological changes characteristic of gestation. Therefore, the aim of this study was to describe the procedure for the extraction and analysis of cortisol in hair samples from pregnant women throughout gestation. **Methods**: Hair samples, three centimeters in length, were obtained from women during the first, second, and third trimesters of pregnancy. These samples underwent a standardized isopropanol washing step, followed by milling in a laboratory mill using zirconium balls of varying diameters. The resulting hair powder was then weighed and subjected to four incubation cycles using HPLC-grade methanol. Cortisol levels were detected using chemiluminescence immunoassay. **Results**: Mean hair cortisol levels were 4.1 μg/L (ng/mL) in the first trimester, 11.5 μg/L (ng/mL) in the second trimester, and 6.6 μg/L (ng/mL) in the third trimester. **Conclusions**: Standardizing the methodology for cortisol extraction improves the reproducibility of results and, in the long term, may support its incorporation into clinical practice as a useful tool for assessing cortisol levels in both pregnant women and the general population, since hair cortisol enables retrospective evaluation of its cumulative exposure over time, approximately on a monthly basis.

## 1. Introduction

Cortisol is a glucocorticoid hormone produced in the adrenal gland, released into the bloodstream, and primarily regulated by the hypothalamic–pituitary–adrenal (HPA) axis [1,2]. Approximately 80–90% of cortisol circulates in the blood flow, the hormone bound primarily to proteins such as corticosteroid-binding globulin (CBG), while 5–10% is weakly bound to albumin. The remaining 3–10% exists in form of free fraction, representing the biologically active form, capable of diffusing into tissues and exerting its physiological effects [3,4].

Cortisol responds to the circadian rhythm in a manner that is characterized by a pronounced physiological increase in the early morning hours, followed by a gradual decrease throughout the day, reaching its lowest levels during the night and early morning [1,2]. This rhythmic pattern is fundamental for maintaining homeostasis in the body, as cortisol participates in the regulation of multiple biological processes, including the metabolism of glucose, proteins, and lipids [3,5,6], as well as in the modulation of the stress response and inflammatory processes [7,8], making it a key hormone in the stress response, whether physical or psychological in its origin [1,2]. The rise in its level allows for a rapid response in case of threat or demand, returning to its normal secretion level once the condition disappears [1,2,3].

However, in situations where the stress is persistent or perceived as overwhelming by the individual, sustained activation of the hypothalamic–pituitary–adrenal (HPA) axis may occur, with alterations in the secretion and regulation of cortisol, which are associated with adverse effects on metabolic, immunological and neurobiological processes, impacting physiological functions [7,8,9,10,11]. This characteristic has led to its classification as a biomarker of hypothalamic–pituitary–adrenal (HPA) axis activity and, depending on the biological samples where its level is measured (e.g., saliva, serum, nail, and hair), as an indicator of acute reactivity or chronic stress exposure. In particular, hair cortisol is considered an emerging biomarker for the assessment of chronic stress load over weeks and/or months [12,13].

It is worth noting that, under states of chronic stress, cells trigger an allostatic state [14,15], characterized by an imbalance of primary chemical mediators and cortisol levels. This fact has been linked to immune pathologies [7,8], mental health conditions such as obsessive–compulsive disorder [16], anxiety, post-traumatic stress, panic disorder, anguish and depression [10,12,17,18,19,20,21,22]. Furthermore, its relationship with obesity, cardiovascular problems, Addison’s disease, Cushing’s syndrome and adrenal insufficiency has been widely established [23,24,25,26,27,28,29].

The diverse functions that cortisol performs in the body lead to the need for further analysis and expansion of studies examining expected values in the population, considering both the techniques used and the different stages of the life cycle of people. This aspect is especially relevant during pregnancy, due to the multiple physiological adaptations that regulate maternal well-being and placental function, which, consequently, affect the health of the offspring [30,31,32].

Unfortunately, measuring cortisol levels has not been without its difficulties, mainly due to its circadian production pattern and the multiple variables that interfere with it [1,2,33,34]. Cortisol levels can be measured at specific time points by determining their presence in blood, plasma, serum, urine, and saliva [9,10,22,35]. Of these methods, salivary sampling is the most commonly used because it is less invasive [10] and easier to collect, as it does not cause stress like venous blood sampling [33]. It is also important to note that in this type of sampling the free cortisol fraction is obtained, a crucial finding since only the free fraction represents biological activity [4].

As a consequence of the above, various processing techniques have emerged with the purpose of reflecting the function of the hypothalamic–pituitary–adrenal (HPA) axis, considering the diversity of the samples. The most traditional are those obtained in serum and 24 h urine [35]; however, they do not adequately capture the dynamic and circadian nature of the HPA axis [1,2], which has driven to the use of alternative matrices to determine the free cortisol fraction such as saliva and hair [10,22,23,24,35].

Different analytical methods can affect sensitivity, specificity, resource availability, and associated costs. Therefore, the selection of the analytical method should consider the study objectives, the type of physiological information required, and the technical feasibility of its implementation [35,36]. Based on this premise, we find that quantification can be performed using immunoassays, such as enzyme-linked immunosorbent assay (ELISA) and chemiluminescent immunoassay (CLIA), which allow for an indirect measurement of cortisol based on the antigen–antibody interaction and the generation of a detectable signal. These methods offer significant advantages, such as automation, speed, lower cost, and wide availability in clinical practice, making them particularly useful tools in both clinical and population studies [35,36]. However, immunoassays, including CLIA, can have important analytical limitations associated with cross-reactivity with other steroids, interference from binding proteins, and lower specificity compared to chromatographic methods. On the other hand, techniques such as high-performance liquid chromatography (HPLC) and liquid chromatography coupled to tandem mass spectrometry (LC-MS/MS) allow for more specific identification of cortisol based on its physicochemical properties, with the latter being considered the gold standard. However, its implementation is considerably limited by its high cost, technical complexity and lower availability, which has favored the use of automated immunoassays such as CLIA. Likewise, emerging technologies such as SPR biosensors offer high sensitivity, although their application is mainly limited to research. Therefore, the selection of the methodology for cortisol obtention involves a balance between analytical precision, accessibility, and operational feasibility [35,36,37].

As previously mentioned, the free cortisol fraction can be estimated by analyzing biological matrices such as hair and nails, providing a retrospective record of chronic cortisol exposure [4,31,35,38]. This is because cortisol diffuses passively from the systemic circulation (capillaries) into keratinized matrices, specifically the hair cortex, where it is retained within the hair shaft during the process of hair formation. This allows for the understanding of long-standing hypercortisolemia [39,40]. Of the two methods mentioned, measurement in hair is the most accurate, as hair grows continuously, at a rate of approximately 1 cm per month, thus enabling retrospective cortisol analysis [41]. For this reason, it is considered a suitable biomarker for the assessment of chronic stress [22,39,40,42]. It is worth noting that the sample can be obtained non-invasively and stored for extended periods wrapped in aluminum foil, without significantly altering the results [43], which facilitates its transport, preservation, and subsequent analysis.

So far, few studies have evaluated cortisol concentration in the hair of pregnant women [31,44,45,46,47], as most of the available evidence is based on saliva or blood analysis [10,20]. However, in this population, it is essential to consider the physiological changes of endocrine regulation during pregnancy, characterized by the progressive production of placental corticotropin-releasing hormone (CRH-p), which induces sustained activation of the HPA axis and a continuous increase in maternal cortisol levels throughout gestation [28,32]. In spite of the growing interest in using hair cortisol as a biomarker of chronic stress, the available evidence presents significant methodological limitations [40,42], especially in the detailed description of extraction, processing, and analysis protocols [48,49,50], which hinders the replicability of studies and the comparison of results between investigations. This methodological heterogeneity has limited the standardization and dissemination of the technique, hindering its potential incorporation into the clinical context.

Taking into consideration the facts mentioned above, the objective of this article is to provide a detailed and reproducible description of a protocol for the extraction and analysis of cortisol in hair, using the chemiluminescence immunoassay method, given that this technology is widely available in clinical laboratories. It is hoped that this proposal will contribute to the evaluation of hair cortisol in pregnant and non-pregnant populations and strengthen its use in the assessment of circadian rhythm disorders and its potential applications in health.

## 2. Materials and Methods

The materials, reagents, and equipment used in the sample collection, washing, grinding, and cortisol extraction stages are described in Table 1.

Hair samples were obtained, after obtaining informed consent, from pregnant women attending various family health centers in the city of Concepción, Chile. This research project was approved by the Ethics Committee of the Concepción Health Service (approval code CEC-SSC:23-11-55). The sample consisted of 114 pregnant women, selected from those without previously diagnosed chronic cardiovascular or immunological conditions, and excluding those who reported alcohol, drug, or prescription medication use. The pregnant women’s admission form also included demographic data such as height and weight, age, gestational age, and obstetric history.

**Figure 1:**
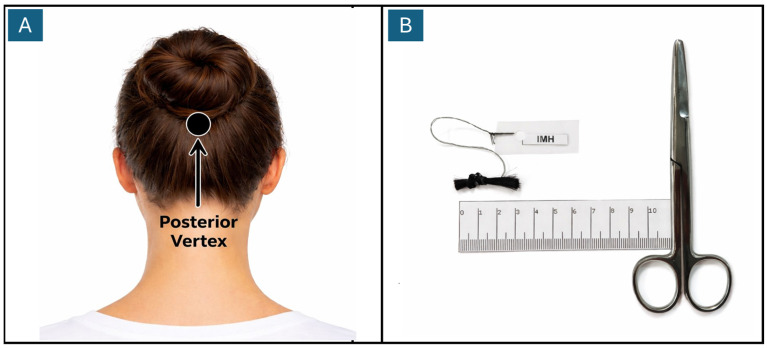
Sample collection. (**A**) A sample of hair was collected from the back of the head, on the posterior vertex region (Arrow). The use of procedure gloves is optional. (**B**) A 3 cm length section of hair was cut for analysis, removing hair distal to the scalp.

A sample of hair was collected from all pregnant women (*n* = 114) during the first trimester of pregnancy, considering for the analysis a length of 3 cm measured from the vertex of the head. All participants entered the study before 14 weeks of gestation, allowing for adequate monitoring of changes throughout each trimester of pregnancy. In the second trimester, 96 samples were collected, while in the third trimester a total of 72 were obtained. Losses to follow-up were due to factors such as (i) changes of place of residence, (ii) spontaneous abortions occurring in the first trimester and (iii) premature births, since the inclusion conditions required no reproductive loss or births before 36 weeks of gestation. Additionally, two samples from the first trimester were lost during analysis due to accidental drops in the processing cups. The steps of the protocol designed for hair cortisol extraction are described in detail below:

A. Collection: Hair samples were obtained from the back of the head, as close as possible to the scalp. Samples of hair were selected from the occipital region that were not exposed in the surface layer (see Figure 1). After cutting, the hair was tied with a silicone band and stored in airtight aluminum bags until processing. The average storage time prior to analysis was 10 months, with the samples kept at room temperature (11–18 °C).

**Figure 2:**
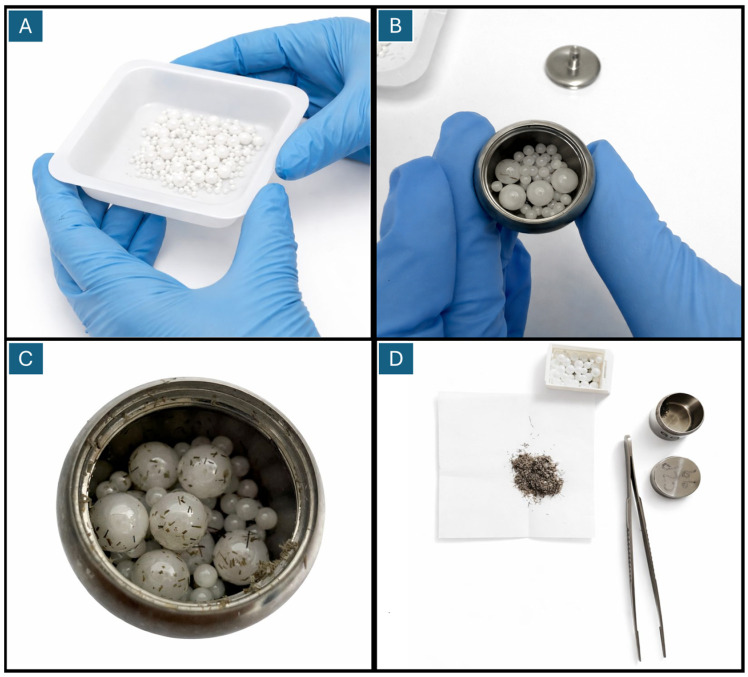
Ball mill grinding. (**A**) Zirconium balls of 2, 3, and 5 mm diameter. (**B**) To maintain sample traceability, each stainless-steel container should hold only one sample. (**C**) A 6 h milling process is recommended to obtain adequate hair powder. (**D**) Sample from one grinding batch obtained from each container.

B. Washing: Without removing the silicone tie, the samples were labeled with the initials of each pregnant woman’s name and subsequently washed by immersion in 15 mL of isopropanol (2-propanol, ≥99.9% purity, Merck, code 1010404000). The procedure consisted of two 15 min wash cycles under constant agitation at 100 rpm on a medium setting. After washing, the samples were dried for 72 h at room temperature, allowing complete evaporation of the isopropanol.
C. Grinding: Each piece of hair was previously cut with sterilized scissors and placed in stainless steel receptacles, which were then placed in a laboratory ball mill (110 V, 2 kg heavy-duty, model TR13749, TR Instruments, Ambala, Haryana, India). Zirconium balls of different diameters were used for grinding, mixed in each receptacle, with a load of between 15 and 20 balls (Figure 2). In each receptacle, groups of 20 samples were processed. After 5 h of operation, a homogeneous hair powder was obtained, suitable for analysis (see Figure 2C). This procedure was performed on samples corresponding to the first, second, and third trimesters of pregnancy.

After grinding, the samples were weighed on an analytical balance, with 30 mg being used for the first test and 60 mg for the second test in the subsequent analysis. They were then stored in 2 mL microcentrifuge tubes, labeled with the initials of each participant, and kept at room temperature (11–14 °C).

D. Extraction: Each sample, previously weighed, was subjected to four washing cycles with HPLC-grade methanol, using volumes of 1 mL, 0.5 mL, 1 mL, and 0.5 mL, respectively, in order to achieve gradual cortisol extraction. The process is described in detail below:

In the first cycle, 1 mL of HPLC-grade methanol was added to each sample. The samples were subsequently vortexed and then continuously agitated at 150 rpm for 1 h. After this process, the samples were placed in a drying oven at 52 °C for 12 h. The following day, the agitation step was continued for 1 h at 150 rpm, followed by centrifugation at 1000 rpm for 1 min. The supernatant, consisting of the first mL of methanol, was transferred to a 1.5 mL microcentrifuge tube and stored at 4 °C, allowing for gradual evaporation of the methanol.

In the second cycle, 0.5 mL of HPLC methanol was added to the residual hair pellet, repeating the previous procedure. The resulting supernatant was mixed with that obtained in the first cycle. This procedure was repeated until all four extraction cycles were completed, collecting the supernatants and storing them at 4 °C. After four days, partial or complete evaporation of the methanol was observed. Throughout the process, the microcentrifuge tubes containing the samples were covered with aluminum foil to protect the hormone from light exposure and thus prevent its degradation.

The final step was the analysis of the evaporated or remaining supernatant. For this, 0.5 mL of PBS (pH 7.4, HyClone, Cytiva) was added, and the samples were vortexed. A volume of 200 µL was extracted using a micropipette for the quantification of cortisol concentration by chemiluminescence immunoassay (CLIA), using the automated MAGLUMI 800 analyzer (Snibe Diagnostics, Shenzhen, China), according to the manufacturer’s instructions. This method is based on a chemiluminescent reaction in which the intensity of the emitted light signal is inversely proportional to the concentration of cortisol present in the sample, determined using commercially available cortisol assay kits validated.

Samples that exceeded the manufacturer’s established detection limit were analyzed in duplicate to confirm the reproducibility of the results. Additionally, pooled sample analyses were performed for each group, resulting in a total of 28 pools, each composed of 8 individual samples, in order to evaluate the group values in relation to the values obtained from the individual samples.

Before loading the samples, the manufacturer’s instructions for proper calibration of the detection kits were strictly followed. Two kits with different detection limits were used due to discontinuity in one of them.

**Figure 3:**
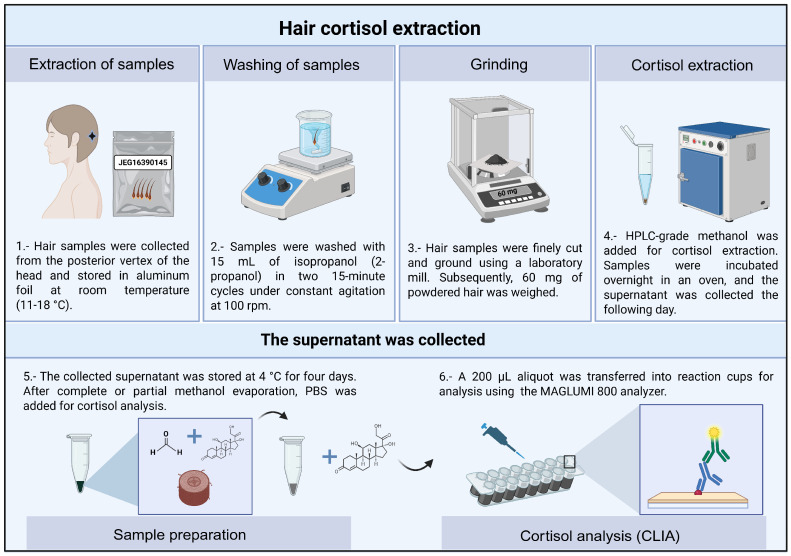
Main stages of the capillary cortisol extraction protocol. The initial stages (1–4) include the extraction, washing, drying, and grinding of the hair samples. Once the extraction stage is complete, the step (5) consists of removing the obtained supernatants. In the final step (6), a 200 µL aliquot of the supernatant was analyzed using the MAGLUMI 800 analyzer based on a chemiluminescent immunoassay (CLIA). Cortisol in the sample competes for antibody binding, and the emitted light intensity is inversely proportional to its concentration.

All the steps followed throughout the cortisol extraction procedure are illustrated and described in Figure 3.

E. Calibration of Cortisol Analysis Kits: Prior to the analyses and in accordance with the manufacturer’s instructions, the cortisol test kits were calibrated on the Maglumi 800 instrument using a master curve system adjusted with assay-specific calibrators, allowing the conversion of the relative light units (RLU) into cortisol concentrations. The accuracy of the method was reported following the Clinical and Laboratory Standards Institute (CLSI EP5-A2) standards [51].

The CLIA method used has adequate analytical sensitivity, with limits of detection (LoB) of 2.5 μg/L (ng/mL) and a measurement range of 2.5 to 600 μg/L (ng/mL). Subsequently, an updated version of the kit was used, which exhibited greater analytical sensitivity, reporting a LoB of 0.5 μg/L (ng/mL), and a limit of quantification (LoQ) close to 4.0 μg/L (ng/mL). The reported analytical range (2–600 μg/L (ng/mL)) allows coverage of physiological and pathological cortisol concentrations.

Intra- and inter-assay precision shows coefficients of variation below 10%, with values even lower than 5% at some concentration levels. Likewise, analytical recovery was found to be within the acceptable range of 90–110%. The evaluation of endogenous and exogenous interferences includes structurally related steroids such as testosterone, progesterone, and androstenedione, as well as hemoglobin, bilirubin, lipids, and biotin. All of these demonstrate an adequate analytical specificity, supporting its applicability.

The results of hair cortisol measurements obtained from hair samples collected during the first, second, and third trimesters of pregnancy were analyzed using univariate statistics to determine differences in the distribution of cortisol values and relative light units (RLU). To analyze the behavior of potential confounding variables, a bivariate statistical analysis was also performed comparing first-trimester cortisol levels with baseline maternal variables, such as maternal age, body mass index, and gestational age at the first hair sample collection. These variables were included due to their potential physiological influence on cortisol concentration during pregnancy.

Due to the fact that the data did not follow a normal distribution, the Friedman test was used to compare cortisol levels across the first, second, and third trimesters of pregnancy. Post hoc pairwise comparisons were performed using the Wilcoxon signed-rank test for paired samples.

Data was tabulated in a Microsoft Excel database and subsequently analyzed using SPSS version 25 statistical software (IBM Corp, Armonk, NY, USA).

## 3. Results

A total of 114 pregnant women, presenting no current chronic or obstetric pathologies, were enrolled in the study before the 14th week of gestation. The average age of the sampled individuals was 29.73 ± 5.23 years. Regarding their biological history, the average weight at the beginning of pregnancy was 72.87 ± 17.99 kg, with an average body mass index (BMI) of 28.66 ± 5.85 kg/m^2^ at the time of enrollment. As for obstetric history, 49.12% were multiparous; of this group, 48.21% had a previous vaginal delivery (Table 2).

The first hair sample was obtained after the participant women began prenatal care (n = 114). The pregnant women had an average gestational age of 11.93 ± 1.85 weeks, and the following capillary cortisol results were obtained: (I) in the first trimester, the average was 4.1 μg/L; (II) in the second trimester, it was 11.5 μg/L; and (III) in the third trimester, it was 6.6 μg/L (Table 3).

The association between body mass index (BMI), calculated from height and weight at the first prenatal check-up, and cortisol levels obtained in the first trimester of pregnancy was evaluated, with an observed *p*-value of 0.278. The relationship between cortisol levels and maternal age, as well as gestational age at the time of the sampling, was also explored, with *p*-values of 0.071 and 0.874, respectively (Table 4).

Cortisol levels varied throughout gestation. Mean hair cortisol concentrations were 3.73 μg/L in the first trimester, 10.39 μg/L in the second trimester, and 6.74 μg/L in the third trimester. Statistically significant differences in cortisol levels were observed across the three trimesters (Friedman test, *p* < 0.001), showing an increase from the first to the second trimester, followed by a decrease in the third trimester.

To determine between which trimesters the differences occurred, post hoc pairwise comparisons were performed using the Wilcoxon signed-rank test for paired samples. Statistically significant differences were observed between all trimester comparisons (first vs. second trimester, first vs. third trimester, and second vs. third trimester), with all comparisons reaching statistical significance (*p* < 0.0001) (see Table 5 and Table 6).

## 4. Discussion

Publications addressing cortisol extraction generally describe similar procedures in their fundamental stages; however, the order in which these procedures are applied, as well as some specific conditions, vary among the different protocols described [39,40,42,48]. The main phases, as well as the considerations taken into account in the present study, are detailed below.

Sample collection was carried out following techniques previously described in the literature. Specifically, samples were taken from the posterior vertex of the head; surgical scissors were used for cutting, and the initial wash was performed with isopropanol [39,40,42]. It should be noted that the literature shows differences regarding the number of washes, ranging from one to two. Our protocol followed the recommendation of Greff et al. (2019), who demonstrated that between one and four wash cycles there are differences in cortisol recovery, reaching up to 95% extraction in the fourth cycle [42]. For this reason, four consecutive washes were used, which allowed us to obtain virtually all of the cortisol present in the sample. Drying was carried out at room temperature, in accordance with previously reported criteria [39,42].

In the milling stage, several authors have described the use of specialized equipment, including cryogenic mills [48]. In our case, a conventional laboratory mill was used, as it is a more accessible alternative in our context, extending the processing time until a homogeneous hair powder was achieved. To ensure traceability, each sample was placed in individual containers, which facilitated organization and identification.

Regarding the homogenization stage, our procedure differs from some previously described protocols [40,42], in which the milling step is performed before hair washing and the weight is determined on the entire hair segment. In the present study, washing was carried out beforehand, and homogenization or milling was performed afterward; once the milled sample was obtained, it was weighed for analysis. This modification was implemented to reduce weight variability, considering that material loss can occur during milling, as variations in weight were observed in preliminary tests by our group using the described methods.

Regarding the reagents, 3 mL of HPLC-grade methanol was used in our study for the extraction. This amount varies among different protocols, with volumes of 2 mL or up to 4 mL reported in others [39,42,48]. We decided to use analytical-grade methanol to achieve low concentrations and reduce the probability of contaminants in the compound [52].

A very important aspect to consider in this process is protecting the supernatant from light exposure. This was achieved by keeping this phase completely covered with aluminum foil throughout the entire process. Although this practice had not been explicitly described in previously published protocols, it constitutes, in our opinion, a good recommendation, since cortisol degrades rapidly and significantly in response to variations in temperature and ambient light [11,39,40]. In this context, it has been considered that free cortisol, diffused and subsequently stored in hair, is particularly susceptible to these conditions [40].

Cortisol concentrations were expressed in ng/mL (μg/L) because quantification was performed on the supernatant obtained after the capillary extraction process. Although most studies report cortisol in hair expressed as sample mass (pg/mg), using ng/mL is appropriate when the measurement is performed directly on the liquid fraction of the extract [40,42], depending on the technique used, the biological sample, the availability of equipment, and the clinical applicability of the method.

In our study, the CLIA method was selected because it allows for rapid, reproducible, and accessible measurement. Although liquid chromatography coupled to tandem mass spectrometry (LC-MS/MS) is considered the gold standard due to its high specificity and analytical sensitivity, its high cost and technical complexity limit its implementation in many clinical contexts as well as in larger scale studies [35,36,37].

Although the CLIA method may have limitations, such as possible cross-reactivity with structurally related compounds and the presence of analytical interferences, advances in the standardization of calibrators and in validation processes have allowed for improvements in its analytical performance [35,36].

In the present study, baseline factors related to cortisol levels were the woman’s age, the gestational age at the time of the first sample collection and the body mass index, characteristics that indicate specific metabolic conditions [53,54,55]. Evidence suggests that these baseline factors can modify cortisol levels, which are generally measured by conventional methods (i.e., serum, urine, and saliva), reflecting the acute release of the steroid [40,42]. When comparing first-trimester cortisol levels measured in hair, no statistically significant differences were found between the described variables.

Regarding hair cortisol analyses, differences were observed between the different trimesters of pregnancy. These variations could be associated with the physiological changes inherent to pregnancy, considering that cortisol undergoes progressive modifications during gestation [44,54]. However, in our study, a decrease in cortisol levels was detected in the third trimester, which differs from what has been described in studies that have evaluated cortisol in other biological matrices, such as blood, saliva, or urine, where a progressive increase in this hormone is usually reported as pregnancy progresses.

A possible explanation for this finding could be related to characteristics of the studied population. In our studied group, a significant proportion of pregnant women performed both paid and unpaid work during pregnancy, while in the third trimester many were on maternity leave, which could influence the reduction in exposure to daily stressors. However, this hypothesis should be interpreted with caution, as the present study was not designed to evaluate psychosocial variables in depth. These results suggest the need for further investigation into hair cortisol levels during pregnancy and their possible relationship with clinical, occupational, and psychosocial factors, in order to better understand the variations observed in this biomarker in pregnant women.

Another factor that may contribute to these differences is related to methodological aspects. In a similar study, hair cortisol was assessed across the three trimesters of pregnancy using a single hair sample collected at delivery, in which the hair length was segmented to retrospectively represent cortisol exposure during each trimester [44,45]. This methodological approach differs from that used in the present study, in which samples were collected independently during each trimester, which may contribute to the differences observed between studies.

Finally, although the use of mass spectrometry for cortisol detection is predominantly reported in the literature [27,48], in this study the chemiluminescence technique was chosen using the Maglumi 800 equipment, considering its greater availability in healthcare and research centers, and also taking into account its potential clinical application while safeguarding the limitations previously described.

## 5. Conclusions

This study describes in detail the procedure for obtaining and analyzing cortisol in hair samples from pregnant women during pregnancy. This technique is of particular interest because the stability of the hair growth rate allows for the retrospective, monthly evaluation of accumulated cortisol levels.

In relation to the protocol described in this work, it is recommended to wash the hair sample prior to the grinding stage, since its final weight can vary by up to 0.30 mg, which constitutes an interference factor in the results, a condition that was verified by our research team.

It is also advisable to store the samples in methanol, given the polar affinity of the steroid hormone for this solvent, which promotes its stability. If the samples are not going to be analyzed immediately, it is important to note that PBS should not be added, as cortisol is highly susceptible to temperature changes and light exposure, factors that directly contribute to its degradation, a fact also confirmed by the research team. It is also recommended to perform the evaporation of methanol under refrigeration at 4 °C, as this allows for a gradual obtaining of cortisol, reducing the possibility of degradation during the process. Regarding the reagent, the use of HPLC-grade methanol as the extraction solvent is recommended due to its high purity and low interfering content. These characteristics improve the reproducibility and reliability of the analytical results when quantifying compounds present in low concentrations, such as steroids.

The results obtained demonstrate an adequate analytical precision of the method, with coefficients of variation within acceptable ranges for clinical immunoassays. These findings are consistent with the manufacturer’s validation, which reports coefficients of variation below 10% and a recovery between 90% and 110%.

Finally, it is considered essential to advance the standardization of samples in order to have a unified protocol that guarantees reproducible and comparable results. The strategy described in this work will allow for the establishment of agreed-upon guidelines for monitoring, diagnosis, and the definition of normality criteria, which in turn will facilitate the extrapolation of this test to clinical practice. However, it is important to consider that for a more in-depth study of the HPA axis, it would be appropriate to complement this technique with other cortisol measurement methods, taking into account the various forms of its biological distribution, as well as combining this technique with measurements of other hormones, to better understand the complex functioning of this circuit.

The extraction protocol used in our investigation allowed for consistent cortisol measurement across the different trimesters of pregnancy, supporting the possibility of using this methodology in this population and the feasibility of optimizing and standardizing this technique for future applications. Furthermore, the availability of the materials and equipment used favors its potential implementation not only in research settings but also in clinical practice.

## Figures and Tables

**Table 1:** Materials, reagents and equipment for hair cortisol extraction.

Collection	Washing	Milling	Extraction
Surgical scissors with rounded tips. Silicone tie. Aluminum foil bag with airtight seal (11 mm × 16 mm × 3 mm). Permanent marker for sample identification. Ruler graduated in millimeters.	15 mL beaker. Orbital stirrer. Isopropanol (≥99.9%, analytical purity).	Stainless steel receptacles (24 mm× 25.5 mm). Zirconium balls (diameters 5, 3, 2.5, and 2 mm). Laboratory mill (110 V, 2 kg capacity, heavy-duty). Analytical balance (0.1 mg precision). Microcentrifuge tubes (2 mL).	HPLC-grade methanol (≥99.9% purity). 1.5 mL microcentrifuge tubes. Vortex mixer. Benchtop centrifuge. Drying oven. Aluminum foil. PBS (phosphate-buffered saline, pH 7.4).

**Table 2:** Demographic characteristics of participating mothers.

Variable	$\bar{x}\pm SD$
Age of pregnant woman (years)	29.73 ± 5.23
Range	19–42
Weight (kg)	72.87 ± 17.99
Range	40.7–99.6
Mother’s height (cm)	1.60 ± 0.063
Range	1.41–1.78
Body Mass Index—BMI (kg/m^2^)	28.66 ± 5.85
Range	16.94 ± 43.6
Variable	n (%)
Primigravida	58 (50.9)
Multigravida	56 (49.1)

Data are presented as n (%) for categorical variables and mean ± s for continuous variables. $\bar{x}$: Mean, SD: standard deviation.

**Table 3:** Distribution of capillary cortisol values recorded in each trimester of pregnancy.

	N	$\bar{x}\pm SD$	$M_{e}$	Min–Max
First Result (μg/L)	112	4.1 ± 4.3	2.5	0.5–30.6
First RLU	112	927,541 ± 112,214	956,644	493,879–1,105,531
Second Result (μg/L)	92	11.5 ± 14.8	10.3	0.5–132
Second RLU	92	753,561 ± 152,842	752,671	152,679–1,078,298
Third Result (μg/L)	69	6.6 ± 4.5	5.5	0.5–18.2
Third RLU	69	844,978 ± 113,004	857,872	594,269–1,080,962
Average Result	67	6.9 ± 3.6	6.6	1.17–13.7
Average RLU	67	843,039.9 ± 81,842.3	853,517.3	685,730.7–1,024,071.7

$\bar{x}$: Mean; SD: standard deviation; M_e_: Median; Min: minimum; Max: maximum. (1 µg/L = 1 ng/mL).

**Table 4:** Correlation between hair cortisol levels and maternal age, BMI, and weeks of gestation.

	Correlation Coefficient	*p*-Value
	Mother’s Age	
First result (μg/L)	−0.021	0.071
First RLU	0.823	0.460
	IMC (kg/m^2^)	
First result (μg/L)	0.106	0.278
First RLU	−0.173	0.076
	Weeks of gestation	
First result (μg/L)	−0.015	0.874
First RLU	−0.031	0.748

Note: Correlation coefficients were calculated using Spearman’s rank correlation test. Statistical significance was set at *p* < 0.05. RLU: relative light units; BMI: body mass index.

**Table 5:** Distribution of hair cortisol levels according to trimester of gestation.

	n	Mean	Standard Deviation	Min.	Max.	*p*-Value	Statistical Test
First result (μg/L)	66	3.7259	3.20949	0.5	13.6	<0.0001	Friedman
Second result (μg/L)	66	10.388	6.82608	0.5	26		
Third result (μg/L)	66	6.7352	4.58668	0.5	18.2		

Note: Data are presented as mean and standard deviation (SD). Cortisol levels are expressed in μg/L (ng/mL). Differences across trimesters were analyzed using the Friedman test. A *p*-value < 0.05 was considered statistically significant.

**Table 6:** Pairwise comparison of hair cortisol levels between trimesters of gestation.

Comparison	*p*-Value	Statistical Test
First result (μg/L)/ Second result (μg/L)	<0.0001	Wilcoxon
First result (μg/L)/ Third result (μg/L)	<0.0001	Wilcoxon
Second result (μg/L)/ Third result (μg/L)	<0.0001	Wilcoxon

Note: Pairwise comparisons were performed using the Wilcoxon signed-rank test for paired samples. Statistical significance was set at *p* < 0.05. Cortisol levels are expressed in μg/L (ng/mL).

## Data Availability

The datasets generated and/or analyzed during the current study are available from the corresponding author upon reasonable request.

## Abbreviations

The following abbreviations are used in this manuscript:

BMI	Body mass index
CBG	Corticosteroid-binding globulin
CLIA	Chemiluminescent immunoassay
CRH-p	Placental corticotropin-releasing hormone
LC-MS/MS	Liquid chromatography–tandem mass spectrometry
ELISA	Enzyme-linked immunosorbent assay
HPLC	High-performance liquid chromatography
HPA	Hypothalamic–pituitary–adrenal
PBS	Phosphate-buffered saline
RLU	Relative light units
